# Impact of maximum Standardized Uptake Value (SUVmax) evaluated by 18-Fluoro-2-deoxy-D-glucose positron emission tomography/computed tomography (^18^F-FDG-PET/CT) on survival for patients with advanced renal cell carcinoma: a preliminary report

**DOI:** 10.1186/1471-2407-10-667

**Published:** 2010-12-03

**Authors:** Kazuhiro Namura, Ryogo Minamimoto, Masahiro Yao, Kazuhide Makiyama, Takayuki Murakami, Futoshi Sano, Narihiko Hayashi, Ukihide Tateishi, Hanako Ishigaki, Takeshi Kishida, Takeshi Miura, Kazuki Kobayashi, Sumio Noguchi, Tomio Inoue, Yoshinobu Kubota, Noboru Nakaigawa

**Affiliations:** 1 Department of Urology, Yokohama City University Graduate School of Medicine, 3-9 Fukuura kanazawaku Yokohama, 236-0004 Japan; 2 Department of Radiology, Yokohama City University Graduate School of Medicine, Yokohama, Japan; 3 Department of Urology, Kanagawa Cancer Center, Yokohama, Japan; 4 Department of Urology, Yokosuka Kyosai Hospital, Yokosuka, Japan; 5 Advanced Medical Research Center, Yokohama City University, Yokohama, Japan

## Abstract

**Background:**

In this era of molecular targeting therapy when various systematic treatments can be selected, prognostic biomarkers are required for the purpose of risk-directed therapy selection. Numerous reports of various malignancies have revealed that 18-Fluoro-2-deoxy-D-glucose (^18^F-FDG) accumulation, as evaluated by positron emission tomography, can be used to predict the prognosis of patients. The purpose of this study was to evaluate the impact of the maximum standardized uptake value (SUVmax) from 18-fluoro-2-deoxy-D-glucose positron emission tomography/computed tomography (^18^F-FDG PET/CT) on survival for patients with advanced renal cell carcinoma (RCC).

**Methods:**

A total of 26 patients with advanced or metastatic RCC were enrolled in this study. The FDG uptake of all RCC lesions diagnosed by conventional CT was evaluated by ^18^F-FDG PET/CT. The impact of SUVmax on patient survival was analyzed prospectively.

**Results:**

FDG uptake was detected in 230 of 243 lesions (94.7%) excluding lung or liver metastases with diameters of less than 1 cm. The SUVmax of 26 patients ranged between 1.4 and 16.6 (mean 8.8 ± 4.0). The patients with RCC tumors showing high SUVmax demonstrated poor prognosis (*P *= 0.005 hazard ratio 1.326, 95% CI 1.089-1.614). The survival between patients with SUVmax equal to the mean of SUVmax, 8.8 or more and patients with SUVmax less than 8.8 were statistically different (*P *= 0.0012). This is the first report to evaluate the impact of SUVmax on advanced RCC patient survival. However, the number of patients and the follow-up period were still not extensive enough to settle this important question conclusively.

**Conclusions:**

The survival of patients with advanced RCC can be predicted by evaluating their SUVmax using ^18^F-FDG-PET/CT. ^18^F-FDG-PET/CT has potency as an "imaging biomarker" to provide helpful information for the clinical decision-making.

## Background

Renal cell carcinoma (RCC) accounts for 3% of all adult cancers [1]. Approximately 30% of patients are diagnosed with metastases and an additional 20-40% of patients develop metastases after radical nephrectomy with curative intent [2,3]. The outcome of patients with metastatic RCC is poor, with a median survival time of 10 to 21 months [4,5]

Classical cytokine therapies have been the only systematic treatments available for advanced RCC for a long time [6-9]. The oncogenic mechanism of RCC has been elucidated and agents that target relevant biological pathways have been investigated. Multiple tyrosine kinase inhibitors (multiple TKIs) targeting vascular endothelial growth factor receptor (VEGFR) such as sunitinib and sorafenib have revolutionized the treatment of RCC [10,11]. Although mammalian target of rapamycin (mTOR) inhibitor was not available in Japan at the time of this study, the efficacies of mTOR inhibitors have been reported [12,13]. These developments have made it necessary to predict the prognosis of individual patients with advanced RCC and to select optimal management. Many clinical risk factors have been proposed, and classifications of patients using these risk factors have been established. The most common classification was proposed by the Memorial Sloan-Kettering Cancer Center group for cytokine-based therapies (MSKCC classification)[14], and modified criteria adapted for the new era of molecular targeting was reported recently and recommended in the National Comprehensive Cancer Network guideline (NCCN classification)[12,15]. However, these classifications are not enough to determine the best treatment selection for an individual patient. Novel biomarkers to predict the prognosis of individual patients are therefore desired.

During the last decade, 18-fluoro-2-deoxy-D-glucose positron emission tomography (^18^F-FDG-PET) emerged as a useful non-invasive tool to evaluate the metabolic status of tumors. Numerous recent studies of various types of malignancies have reported an association between the ^18^F-FDG accumulation rate evaluated by PET and patient prognosis. The standardized uptake value (SUV) is a semiquantitative simplified measurement of the tissue FDG accumulation rate, and studies of the head-and-neck, lung, and cervical cancer have explored the prognostic significance of the maximum standardized uptake value (SUVmax) [16-19]. However, the role of the SUVmax as a prognostic factor for patients with advanced RCC has not yet been evaluated. In the present study, we evaluated prospectively the impact of SUVmax on the survival of patients with advanced RCC.

## Methods

### Patients

This was a prospective study to clinically follow enrolled patients planning to undergo systematic therapies for advanced RCC. In principle, the pathologies of enrolled cases were confirmed by prior nephrectomy or biopsy, but only one case was diagnosed clinically by conventional imaging because the patient wished to be treated immediately and did not consent to biopsy. The patients were initially assessed by conventional imaging techniques (computed tomography [CT], magnet resonance imaging [MRI], or bone scintigraphy) and diagnosed as stage IV or metastatic RCC. Patients with uncontrolled diabetes mellitus, with other known malignancies and treated with therapeutics the last 2 weeks before the scan were excluded.

The study protocol was approved by the Yokohama City University Institutional Review Board. Written informed consent was obtained from all patients. The patients underwent various therapeutic interventions decided before the evaluation by PET/CT at Yokohama City University Hospital and Kanagawa Cancer Center.

### Imaging

Patients fasted for at least 6 hours prior to intravenous injection of [^18^F] FDG. PET/CT images were obtained using a PET/CT system (Aquiduo 16; Toshiba Medical Systems, Tokyo, Japan). PET/CT images were acquired from the top of the head to the mid thigh at 60 min after intravenous injection of 2.5 MBq/kg of [^18^F] FDG. A low-dose non-contrasted CT scan was acquired first and used for attenuation correction. Emission images were acquired in 3-dimensional mode for 2 min per bed position. After PET acquisition, CECT was performed with a 2-mm slice thickness, 120 kV, 400 mA, 0.5 s/tube rotation, from the top of the head to the mid thigh, with breath holding. A total of 100 ml contrast medium (iopamidol) was administered intravenously at a rate of 1.0 ml/s. The scan delay was set at 120 s after starting the injection of contrast material. Images were reconstructed by attenuation-weighted ordered-subset expectation maximization (OSEM) (four iterations, fourteen subsets, 128' 128 matrix, with 5-mm Gaussian smoothing). The highest SUV in all RCC tumors of each patient was defined as SUVmax. To obtain the SUVmax, the SUV values of all lesions in tumors diagnosed as RCC by CT imaging were analyzed.

### Statistical analysis

Survival time was calculated from the date of evaluation by ^18^F-FDG PET/CT to the date of death. Cox proportional hazards model was used to assess the effects of SUVmax on survival. The cancer-specific survival curve was estimated by the Kaplan-Meier methods, and the resulting curves were compared using the log-rank test. All statistical analyses were carried out with SPSS software (SPSS, Inc, Chicago, IL).

## Results

### Patient characteristics and intervention

A total of 26 patients (21 males and 5 females) were enrolled in this study between 2008 Jun and 2009 October (Table 1). The median age was 61 years (range of 32-82). There were 17 patients with recurrent diseases and 9 with stage IV disease. Pathological examination showed 18 cases of clear cell carcinoma, 5 of papillary, and 2 of clear/sarcomatoid; in one case, the pathological type was unknown. As for prior surgeries, 19 patients had undergone nephrectomy and 4 metastatectomy. Thirteen patients had not undergone previous systematic therapies. Of the other 13 cases with previous systematic therapies, 9 patients had undergone interferon-alpha (IFN-α therapies, one sorafenib, one S1, one combined therapy with IFN-α and sorafenib, and one combined therapies with IFN-α and UFT.

**Table 1:** Characteristics of the 26 patients

Patient ID	Sex	Age	Pathology		Nephrectomy	MSKCC classification	NCCN classification	Prior therapy	SUVmax	SUVmax site
			type	grading						
2	M	63	sarc/clear	3	Yes	Poor	Poor	IFN	15.2	local recurrence
3	M	73	clear	2	Yes	Favorable	Not Poor	IFN IL	8.2	lung
4	M	61	papillary	3	No	Intermediate	Poor	non	8.8	primary
5	F	72	clear	1	No	Intermediate	Not Poor	non	5.2	primary
6	F	55	clear	2	No	Intermediate	Not Poor	IFN IL	6.8	primary
7	M	57	papillary	2	Yes	Intermediate	Poor	N	4.0	bone
8	M	59	clear	2	Yes	Intermediate	Poor	non	7.4	bone
9	M	68	clear	3	Yes	Intermediate	Not Poor	IFN	5.7	bone
10	F	57	clear	2	Yes	Poor	Poor	IFN N	9.1	lymph node
11	M	75	clear	2	Yes	Intermediate	Not Poor	IFN	5.3	muscle
12	M	58	clear	3	Yes	Favorable	Not Poor	IFN	8.5	local recurrence
13	F	61	clear	2	Yes	Intermediate	Poor	IFN C	4.3	pancreas
14	M	59	clear	2	Yes	Intermediate	Not Poor	non	1.4	lung
15	M	61	clear	2	Yes	Intermediate	Poor	IFN	7.7	lymph node
16	M	73	clear	2	No	Poor	Poor	non	16.6	primary
17	F	32	papillary	3	Yes	Favorable	Not Poor	non	16.1	uterus
18	M	56	papillary	2	Yes	Intermediate	Not Poor	C	7.0	lung
19	M	68	clear	2	Yes	Intermediate	Poor	non	9.0	bone
20	M	61	clear	2	Yes	Intermediate	Not Poor	non	5.6	IVC thrombus
21	F	56	sarc/clear	3	Yes	Intermediate	Poor	IFN	10.0	contralateral kidney
22	M	62	clear	3	No	Poor	Poor	non	12.0	primary
23	M	61	clear	3	No	Poor	Poor	non	14.3	primary
24	M	82	clear	1	Yes	Intermediate	Poor	non	5.1	bone
25	M	69	papillary	3	Yes	Favorable	Not Poor	non	13.4	lymph node
26	M	66	clear	1	Yes	Intermediate	Not Poor	IFN	8.2	lung

After the evaluation by PET/CT, 20 patients were treated with multiple TKIs (9 sorafenib, 9 sunitinib, 2 sequential therapy with sorafenib and sunitinib, and 1 sequential therapy with sorafenib and IFN-α), and 6 patients underwent cytokine therapies. At the follow-up end (January 2010), there were 9 cases with cancer death, and we confirmed the other 17 patients alive. There were no cases with death due to other causes and no cases dropped out during follow-up. The median follow-up period was 262 days (range, 43 to 531 days).

### Accumulation of FDG in the lesions diagnosed as RCC tumor by CT imaging

**Figure 1:**
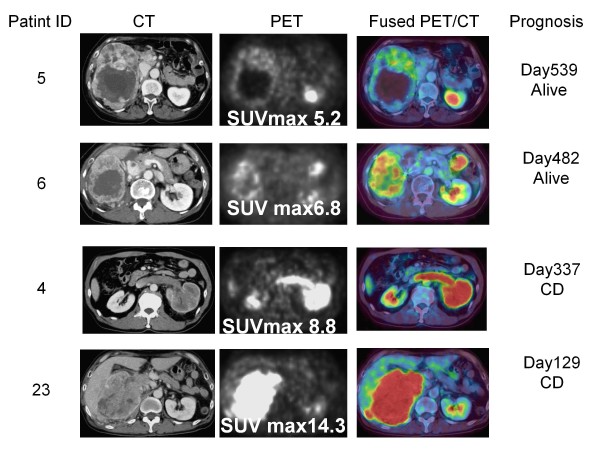
**Four Cases with advanced RCC which original sites showed the highest value of SUV among all RCC sites and their prognosis**. The patients with advanced RCCs having high values of SUV max demonstrated poor clinical courses. SUVmax, maximum standardized uptake value; CT, computed tomography; PET, positron emission tomography; Fused PET/CT, fusion of PET and CT.

**Figure 2:**
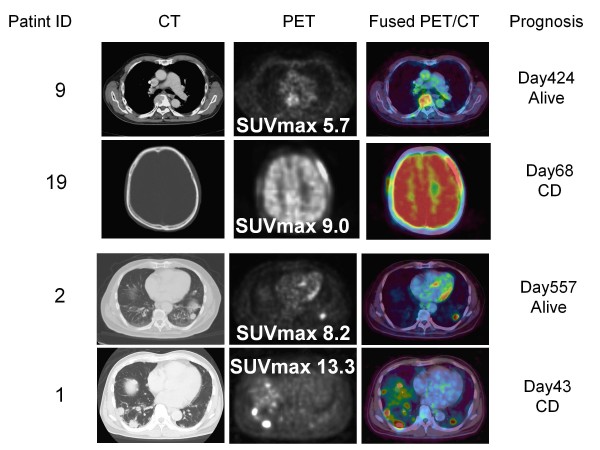
**Four Cases with advanced RCC which metastatic sites showed the highest value of SUV among all RCC sites and their prognosis**. A cranial bone metastasis showed the highest SUV among all RCC sites in Patient 9. A metastasis in thoracic vertebra did in Patinet 19. Lung metastases did in Patient 1 and Patient 2. The patients with advanced RCCs having high values of SUV max demonstrated poor clinical courses. SUVmax, maximum standardized uptake value; CT, computed tomography; PET, positron emission tomography; Fused PET/CT, fusion of PET and CT.

We first, examined the FDG accumulation in all 368 tumor lesions in 26 patients who were diagnosed as stage IV or metastatic RCC by CT imaging. FDG uptake was detected in 230 of 243 lesions (94.7%) excluding lung or liver metastasis with diameters less than 1 cm. On the other hand, among 125 lung or liver lesions with diameters between 5 mm and 9 mm, FDG accumulations were detected in only 21 lesions (16.8%). The SUV in RCC lesions demonstrated various values from undetectable levels to 16.6. In 6 of 7 patients without prior nephrectomy, the primary tumor demonstrated the highest SUV in all RCC tumor lesions (Figure 1), and lung metastasis showed the highest SUV in another. In 19 cases with metastases or recurrence after nephrectomy, bone metastasis demonstrated the highest SUV in 5 cases, lung metastasis in 4 cases, lymph node metastases in 3 cases, and local recurrence in 2 cases (Figure 2). The uterus, pancreas, Inferior Vena Cava thrombus, muscle metastasis, and contra-lateral kidney metastasis demonstrated the highest SUV in one case each.

### The impact of SUVmax on patient survival time

**Figure 3:**
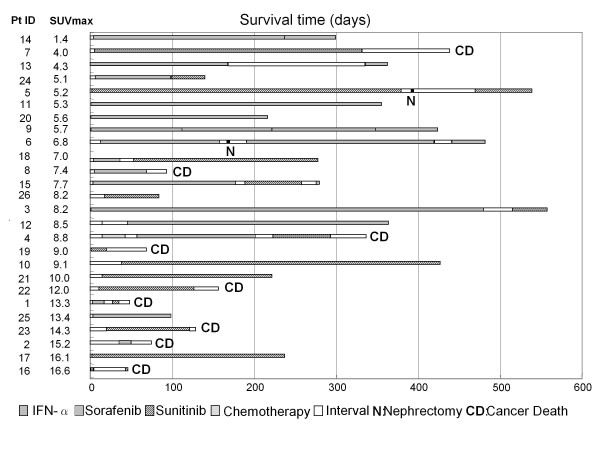
**The treatments and prognoses of 26 patients lined up in order of SUVmax**.

**Figure 4:**
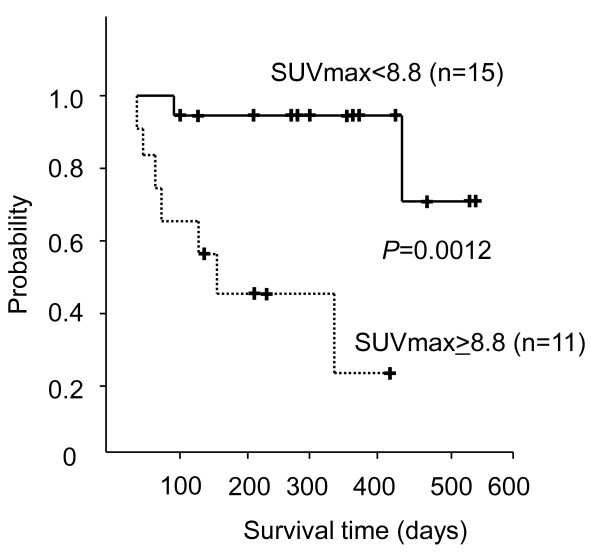
**Survival curves of 26 patients that are stratified by SUVmax of ^18^F-FDG-PET/CT**.

We next analyzed the association between SUVmax and patient survival time. The SUVmax of all patients ranged between 1.4 and 16.6 (mean 8.8 ± 4.0). The patients with RCC tumors showing high SUVmax tended to demonstrate poor prognosis, as shown in Figure 1, 2, 3 (26 patients were lined up in order of SUVmax in Figure 3). When the patient population was subdivided using the mean SUVmax (8.8), only 2 (13%) of 15 patients with RCC tumors having an SUVmax less than 8.8 were dead due to cancer and the median survival time of the 15 patients was not calculated because the number of dead patients was less than half, whereas 7 (64%) of 11 patients RCC tumors having SUVmax equal to 8.8 or more and the median survival time of the 11 patients was 156 day (95% CI 33-279). The survival for these patient subgroups were significantly different (Figure 4) (*P *= 0.0012). When SUVmax was analyzed as a continuous variable, it was correlated with survival time (*P *= 0.005 hazard ratio 1.326 95% CI 1.089-1.614).

## Discussion

In the present study, we demonstrated that the SUVmax evaluated by ^18^F-FDG-PET/CT is a useful predictive "imaging biomarker" for survival of patients with advanced RCC. PET has not been generally used for the screening of RCC due to the urinary excretion of the radiotracer, which can mask the presence of primary lesions [20,21]. However the large RCCs often presenting in stage IV could be evaluated without the influence of urinary excretion of the radiotracer by PET/CT providing combined morphological and functional information (Figure 1). In this study, 7 primary RCC lesions, with diameters ranging from 8.5 cm to 14.7 cm, were examined by ^18^F-FDG-PET/CT, and abnormal FDG accumulations sufficient to evaluate SUV were detected in all lesions. Pathological diagnosis was confirmed in 6 cases. Distant metastases of RCC could also be detected without interference of excretory radiotracers. We did not confirm the pathologies of the individual metastatic lesions, but the previous report by Majhail *et al*. warranted the accuracy of metastasis diagnosis by ^18^F-FDG-PET. They performed biopsy or surgical resection of 36 distant metastatic lesions in 24 patients that were diagnosed by ^18^F-FDG-PET, and pathological findings revealed metastatic RCC in 33 lesions (89%) [22]. In this study, FDG accumulation was evaluated in 94.9% of all RCC lesions diagnosed by CT scan except for lung or liver metastases less than 1 cm. These results were consistent with a previous report [23] and indicated that the information gained by ^18^F-FDG-PET/CT was sufficient to characterize advanced RCCs.

In this era of molecular targeting therapy when various systematic treatments can be selected, prognostic biomarkers are required for the purpose of risk-directed therapy selection. We revealed that the SUVmax has the potency as a novel biomarker to predict the survival time of patients with advanced RCC, by multivariate analyses with standard risk factors or risk classifications. FDG accumulation is thought to be indicative of the metabolic activity of a targeted lesion and it has been found to be a useful index in a variety of cancers. It is reasonable that a tumor with high metabolism would show rapid progression and a poor prognosis. It has been reported recently that ^18^F-FDG PET/CT is useful for evaluating the response to sorafenib and sunitinib treatment of RCC [24,25]. The results showing that these therapeutics decrease the FDG accumulation of RCC lesions encourage the hypothesis that the FDG accumulation is indicative of the biological activity of RCC. Additionally, it has been reported that intratumoral neutrophils were detected in RCCs showing poor prognosis [26]. SUV may reflect not only the biological activity of cancer cells but also the presence of migrating neutrophils.

To our knowledge, this is the first report to evaluate the impact of SUVmax on survival of patients with advanced RCC. However, the number of patients and the follow-up period were limited. Enrollment for this study continues now, and the impact of SUVmax on the survival of patients with advanced RCC will be more apparent from results from an expanded number of patients and follow-up period.

## Conclusions

These preliminary data indicate that the SUVmax evaluated by ^18^F-FDG-PET/CT has an impact on survival in patients with advanced RCC. Additional study with an expanded number of patients and period of follow-up is necessary.

## Conflicts of interests

The authors declare that they have no competing interests.

## Authors' contributions

Noboru Nakaigawa had full access to all the data in the study and takes responsibility for the integrity of the data and the accuracy of the data analysis. All authors read and approved the final manuscript.

Study concept and design: KN, MY, TI, YK, NN

Acquisition of data: KN, RM, KM, NH, TM, FS, UT, KK, SN, HI, TK, TM

Analysis and interpretation of data: KN, MY, NN

Administrative, technical, or material support: TI, YK, NN

Drafting of the manuscript: KN

Critical revision of the manuscript for important intellectual content: MY

Obtaining funding and supervision: NN

## Pre-publication history

The pre-publication history for this paper can be accessed here:

http://www.biomedcentral.com/1471-2407/10/667/prepub
